# Synthetic Bio-Graphene Based Nanomaterials through Different Iron Catalysts

**DOI:** 10.3390/nano8100840

**Published:** 2018-10-16

**Authors:** Qiangu Yan, Jinghao Li, Xuefeng Zhang, Jilei Zhang, Zhiyong Cai

**Affiliations:** 1 Department of Sustainable Bioproducts, Mississippi State University, Starkville, MS 39762, USA; yanqiangu@gmail.com (Q.Y.); xz210@student.exchange.msstate.edu (X.Z.); 2 Forest Products Laboratory, USDA Forest Service, Madison, WI 53726, USA; jli@fs.fed.us

**Keywords:** kraft lignin, catalytic thermal decomposition, carbon-based nanomaterials, iron(III) nitrate, iron(II) sulfate, iron(II) chloride, iron(III) chloride

## Abstract

Kraft lignin was catalytically graphitized to graphene-based nanostructures at 1000 °C under argon atmosphere with four iron catalysts, iron(III) nitrate (Fe-N); iron(II) chloride (Fe-Cl_2_); iron(III) chloride (Fe-Cl_3_); and iron(II) sulfate (Fe-S). The catalytic decomposition process of iron-promoted lignin materials was examined using thermalgravimetric analysis and temperature-programmed decomposition methods. The crystal structure, morphology and surface area of produced materials were characterized by means of X-ray diffraction, Raman, scanning electron microscopy, high resolution transmission electron microscopy and N_2_ adsorption−desorption techniques. Experimental results indicated that iron nitrate catalyst had better iron dispersion three other iron salts. Iron nitrate was the most active catalyst among four iron salts. The low activity of iron in iron chloride-promoted samples was because the residual chlorine over iron surfaces prevent iron interaction with lignin functional groups.

## 1. Introduction

Lignin is a substance in the cell walls of plants that strengthens their structure. During paper pulping processes, lignin is produced as a byproduct. Approximately 50–70 million tons of kraft lignin and 1.8 million tons of lignosulfonates are produced worldwide annually and only about 2% of this amount are utilized for the production of value-added products [1]. Lignosulfonates have a wide variety of applications such as dispersing pesticides, dyes and carbon black; making concrete as plasticizers and fine chemicals as a feedstock [2]. The majority of kraft lignin is used as a low-grade fuel burned on site during kraft pulping operations [2]. Researchers have been trying to valorize lignin waste streams to useful chemicals or materials for large-scale industrial applications.

Lignin contains approximately 60% carbon and can be a carbon precursor for producing templated carbons [3], carbon fibers [4] and activated carbon [5]. A process has been developed to turn kraft lignin into carbon-based nanostructures, that is, graphene-encapsulated nanoparticles [6,7] and graphene materials [8,9]. Kraft lignin was catalytically converted to these carbon-based nanomaterials at high temperature under different atmospheres [9]. Transitional metals such as iron, copper, nickel and molybdenum as catalysts were very active for graphitization of solid carbon materials like kraft lignin [10,11] but their chemical compounds can have different catalytic activities. The catalytic activity of any transitional metal in a solid carbon material is governed by its oxidation state and active specific area, that is, its dispersion in the solid carbon material. It is also affected by the poison species in the reactants, that is, a metal catalyst can be deactivated by poisoning species like sulfur, halides and other residual impurities. On the other hand, the starting state of an active metal can play a significant role in the catalytic performance [12]. The substitution of a transitional metal using its chemical compound to a solid carbon source, as these two are mixed to form a precursor, can lead the formation of different metal phases in terms of different particle sizes and therefore can influence the surface atomic composition of the metal as a catalyst.

Therefore, it is desirable to investigate the effects of different chemical compounds containing the same transitional metal mixed with kraft lignin as precursors on catalytic graphitization of kraft lignin as a carbon in order to find the optimum method for preparing efficient catalysts. Specifically, in this study, the effects of four iron salts, iron nitrate (Fe-N), iron(II) chloride (Fe-Cl_2_), iron(III) chloride (Fe-Cl_3_) and ferrous sulfate (Fe-S) as metal catalyst sources on catalytic carbonization of kraft lignin were investigated.

## 2. Experimental

### 2.1. Iron-Lignin Precursor Preparation

Kraft lignin used in this study as a carbon source was BioChoice Lignin supplied by Domtar Corp., Plymouth, NC, USA. Four analytical grade iron salts, iron(III) nitrate; iron(II) chloride; iron(III) chloride; and iron(II) sulfate purchased from Sigma-Aldrich, Inc. (St. Louis, MO, USA), were used in this study as metal catalyst sources. These iron salts have different physical-chemical properties like solubility and thermal stability [13]. Iron-lignin precursors with their mass ratio of 1/9 (1 part iron and 9 parts lignin) were prepared at room temperature using a co-precipitation technique. Specifically, lignin solution was prepared by adding 300 g of kraft lignin to 300 mL tetrahydrofuran (THF) in a 2000-mL glass beaker and stirring the mixture for 2 h. Iron nitrate solution was prepared by adding 246.0 g of iron(III) nitrate nonahydrate to 100 mL DI water in a 500-mL glass beaker and stirring the mixture until the metal salt dissolved completely. Prepared iron nitrate solution drop-like (~2 mL/min) was added to the lignin solution and the lignin-iron nitrate mixture was stirred for 2 h. The final iron-lignin mixture was kept at room temperature for 24 h and then transferred to an oven and dried at 110 °C for one day. After drying process, a solid iron-promoted lignin mixture as a precursor was obtained. The same mixing and drying procedure was performed to other three iron salts, iron(II) chloride, iron(III), iron(III) chloride and iron(II) sulfate.

### 2.2. Catalytic Graphitization

For each thermal treatment run, 15 g of an iron-promoted lignin precursor were packed in the middle of a 1-inch OD ceramic tubular reactor (McDanel Advanced Ceramic Technologies LLC, Beaver Falls, PA, USA). The carrier argon (99.9% purity) gas was first introduced into the reactor at a flow rate of 50 mL/min for 30 min. The reactor was heated at a rate of 10 °C/min to 1000 °C and kept at 1000 °C for 1 h. The furnace was cooled down by 10 °C/min to room temperature.

### 2.3. Thermogravimetric Analyses (TGA)

TGA analyses of iron-promoted lignin precursors were carried out in a TGA (Shimadzu TGA-50H, Columbia, MD, USA) through isothermal analyses. For each run, 10 mg of iron-promoted lignin mixtures were loaded with argon (99.99% purity, 50 mL/min) gas flowing through the TGA at 50 mL/min as temperature was ramped at 10 °C/min. Three replicates were evaluated for each of four iron-promoted lignin precursors.

### 2.4. Temperature-Programmed Decomposition (TPD) Analyses

TPD analyses of raw kraft lignin and iron-promoted lignin precursors were carried out using an Autochem II 2920 system connected to an on-line residue gas analyzer (Hidden QGA, Peterborough, NH, USA) measuring volatile species. The signals from the mass spectra of 2, 15, 28, 31, 34, 44, 78 and 94 (*m*/*z*) were identified as major contributors from s evolved gases and volatiles like H_2_, CH_4_, CO, CH_3_OH, H_2_S, CO_2_, benzene and phenol. Gaseous species from thermal decomposition of iron-promoted lignin precursors were examined, that is, HCl (*m*/*z* = 36) from FeCl_2_ and FeCl_3_ samples, NO_2_ (*m*/*z* = 46) and O_2_ (*m*/*z* = 32) from Fe (NO_3_)_3_ samples and SO_2_ (*m*/*z* = 64) and SO_3_ (*m*/*z* = 80) from FeSO_4_ samples.

### 2.5. Elemental Analyses

Carbon (C), hydrogen (H), nitrogen (N) and sulfur (S) contents in raw kraft lignin and thermally treated iron-promoted lignin precursors were measured by a PerkinElmer PE 2400 CHN Elemental Analyzer (Billerica, MA, USA). At least three measurements were conducted for each sample. Chloride was analyzed by dissolving samples in sulfuric acid and by distillation with titration of the distillate according to the Volhard-Charpenter method [14].

### 2.6. Characterization

X-ray powder diffraction (XRD) patterns of thermally treated iron-promoted lignin precursors were obtained using a Rigaku Ultima III X-ray Diffraction System operated at 40 kV and 44 mA using Cu-Kα radiation with a wavelength of 15406 Å, from 15° to 80° at a scan rate of 0.02 °s^−1^. The Jade powder diffraction analysis software from Materials Data, Inc. (Livermore, CA, USA) was used for both qualitative and quantitative analyses of polycrystalline powder materials. The mean particle size, L, was calculated for the most intense diffraction peaks of α-Fe, γ-Fe and Fe_3_C using the Scherrer formula [15,16]. The surface areas of thermally treated iron-promoted lignin precursors were determined using N_2_ adsorption−desorption (Quantachrome, Autosorb-1). Prior to measurements, all samples were degassed at 300 °C for 3 h.

The morphology of a thermally treated iron-promoted lignin precursor was investigated using a Scanned Electron Microscope (SEM) (Peabody, MA, USA). All SEM samples were pre-coated with 10 nm Pt before introduced into the vacuum chamber. The system was operated with an accelerating voltage of 10 kV. The particle sizes of thermally treated iron-promoted lignin precursors were examined using a JEOL JEM-100CX II Transmission Electron Microscope (TEM, Peabody, MA, USA) operated at an accelerating voltage of 200 kV. All TEM samples were sonicated in ethanol solution for 1 min before transfer to copper grids.

Raman spectra of thermally treated iron-promoted lignin precursors were obtained on a Jobin-Yvon microspectrometer (Edison, NJ, USA) equipped with an excitation laser source emitting at 514 nm and having an incident power around 1 mW on a thin surface. Twenty spectra were collected for each sample. Deconvolution of the spectra was performed with the assumption of mixed Gaussian/Lorentzian peaks describing both main D- and G-bands and two minor ones. The D, G and 2D peaks were fitted with Lorentz functions. The I_D_/I_G_ ratio was calculated using the heights of D and G peak intensities. The crystalline size along the a-axis (L_a_) was calculated using the Cançado equation [17].

## 3. Results and Discussion

### 3.1. Thermal Analyses

**Figure 1:**
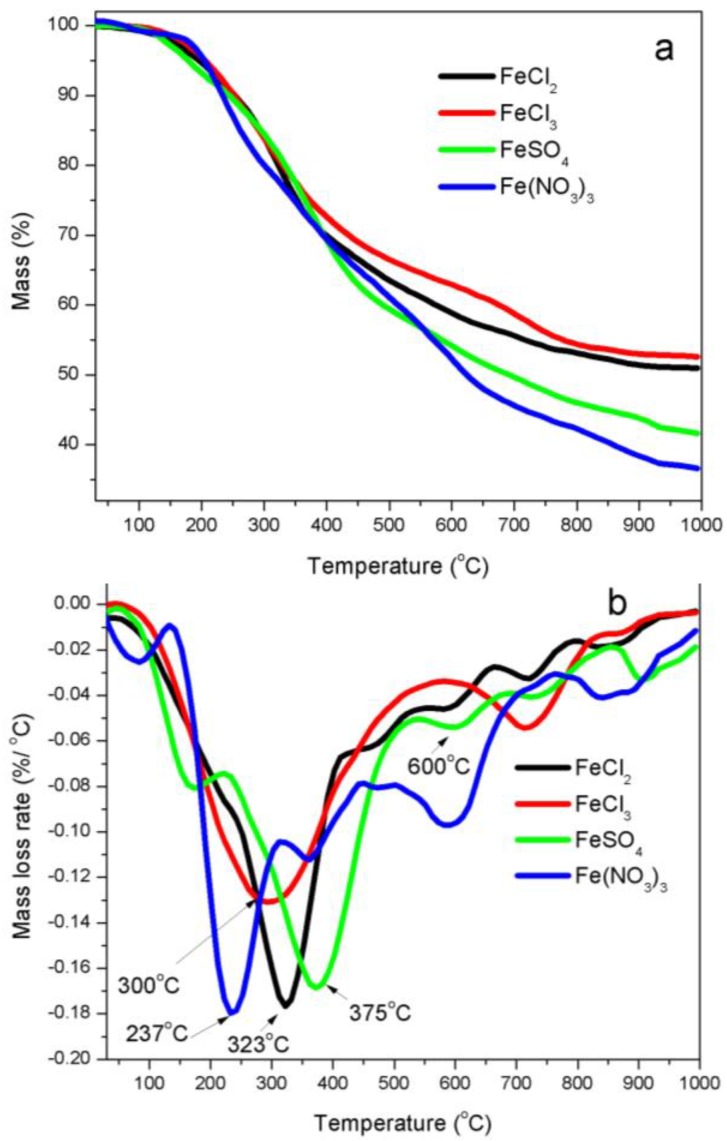
TG (**a**) and DTG (**b**) curves of kraft lignin promoted with four iron salts: FeCl_2_, FeCl_3_, FeSO_4_ and Fe (NO_3_)_3_.

***Thermogravimetric analyses (TGA)***—FTIR spectra demonstrated that there were many oxygen-containing (carboxylic, carbonyl, acyl, alkoxyl, ketones, esters and ethers, et al.), alkyl and aromatic functional groups in kraft lignin [18]. These functional groups in kraft lignin were broken down or cracked with increasing of temperature during the thermal process. Figure 1 shows TG and DTG curves of four iron salts promoted lignin precursors. Figure 1a shows that solid residues as percentage of starting weights of iron-promoted lignin precursors after decomposition were 52.6%, 50.9%, 41.6% and 36.6% for iron salts FeCl_3_, FeCl_2_, FeSO_4_ and Fe (NO_3_)_3_, respectively, which indicates that the catalytic graphitization activity of four iron catalysts on kraft lignin materials was in ascending order FeCl_3_ < FeCl_2_ < FeSO_4_ < Fe (NO_3_)_3_.

The thermal decomposition process of iron-promoted lignin precursors can be divided into four stages (Figure 1b). The first stage is characterized by a mass loss because of the evaporation of surface moisture and dehydration of combined moistures from iron-promoted lignin precursors. The second stage corresponded to the de-polymerization of kraft lignin and decomposition of iron species. During the de-polymerization process, the oxygen-containing groups in alkyl side chains of lignin basic units were catalytically decomposed. The mass decreased rapidly due to the breakage of large number of ether and C–C bonds connected on phenyl propane units, which generated small-molecule gases and macromolecular condensable volatiles. The maximum rates of these weight losses occurred at the temperatures of 323 °C, 300 °C, 375 °C and 237 °C for FeCl_2_-, FeCl_3_-, FeSO_4_- and Fe (NO_3_)_3_-promoted lignin precursors, respectively. The third mass loss (Figure 1b), corresponding to decomposition of kraft lignin char yielded after the completion of the second stage, indicated that the functional groups of kraft lignin continued to decompose as the temperature increased, which led to the aromatization of kraft lignin char matrix. The fourth mass loss stage was characterized with a further carbonization and graphitization process of chars in a wider temperature range up to 1000 °C, where the mass loss was mainly because of the decomposition of phenols, ether and C–H groups of kraft lignin chars, which released out CO and H_2_ as main gases.

***Temperature-programmed decomposition (TPD) analyses***—Lignin is an across-linked type macromolecule and mostly formed via free radical coupling of three basic hydroxyphenylpropanoid monolignols: coumaryl, coniferyl and sinapyl alcohols [19]. With the aid of catalysts, oxidative agents, or thermal treatment, lignin will break down at positions 1, 4, 5 and β [18], accompanying the release of incondensable gases like H_2_, CO_2_, CO, CH_4_, C_2_H_6_, C_2_H_4_, H_2_S, trace amounts of gaseous organics (CH_3_OH, C_6_H_6_OH) and water vapor or volatile products. In addition, gaseous species are produced from the thermal decomposition of iron salts such as HCl from Fe-Cl_2_ and Fe-Cl_3_, NO_2_ and O_2_ from Fe-N and SO_2_ and SO_3_ from Fe-S.

**Figure 2:**
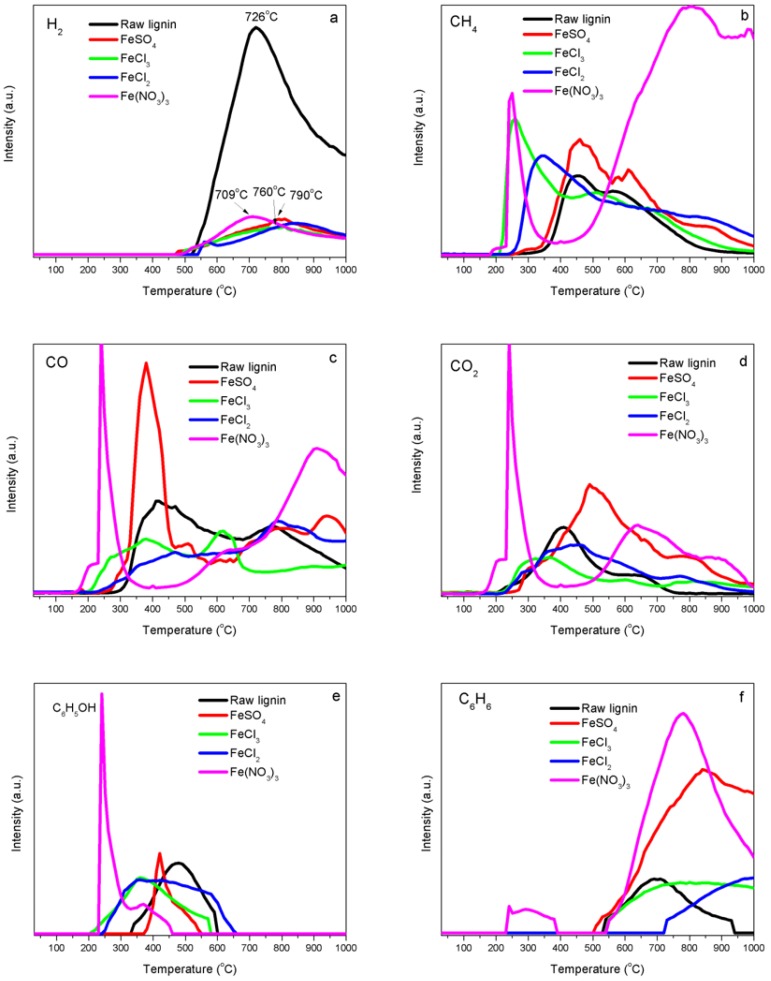

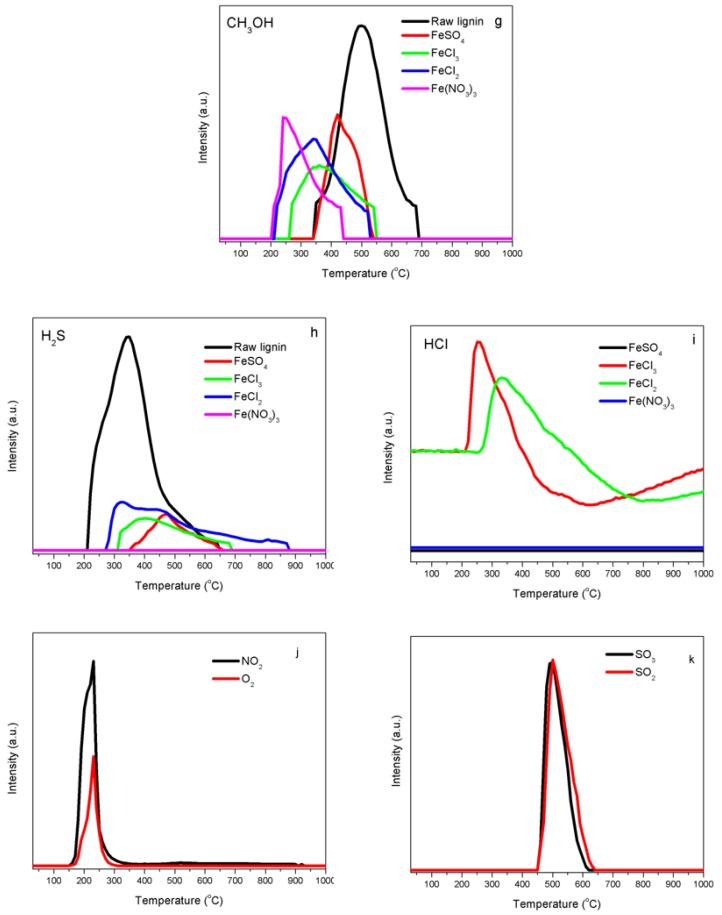
Evolution of gases and volatiles from thermally treated kraft lignin samples promoted with different iron compounds (FeCl_2_, FeCl_3_, FeSO_4_ and Fe (NO_3_)_3_): (**a**) H_2_, (**b**) CH_4_, (**c**) CO, (**d**) CO_2_, (**e**) phenol, (**f**) benzene, (**g**) CH_3_OH, (**h**) H_2_S, (**i**) HCl (from FeCl_2_, FeCl_3_), (**j**) NO_2_ and O_2_ (from Fe (NO_3_)_3_), (**k**) SO_2_ and SO_3_ (from FeSO_4_).

Figure 2 shows the evolution curves of release intensities of various gaseous species as a function of heating temperature recorded during the temperature-programmed decomposition of raw kraft lignin and iron-promoted lignin precursors. For raw kraft lignin, the releasing of H_2_ began at 520 °C and reached its maximum value at 726 °C. For Fe-N, the releasing of H_2_ started at 466 °C and reached its maximum value at 709 °C. H_2_ released from Fe-S at 466 °C and reached its maximum value at 790 °C. Fe-Cl_3_ samples began its H_2_ release at 493 °C with a maximum value reached at 810 °C, while Fe-Cl_2_ samples began its H_2_ release at 537 °C with a maximum value reached at 840 °C. More hydrogen was supposed to be produced from the samples with iron catalysts and evolution temperature were supposed to be lower than raw kraft lignin samples. However, the evolution peaks were weaker and flatter and the hydrogen peaks for FeSO_4_, FeCl_3_ and FeCl_2_ shifted to the higher temperatures. This was caused by the reverse water-gas shift reaction (RWGSR) (CO_2_(g) + H_2_(g) = CO(g) + H_2_O(g)). Part of hydrogen from the thermal cracking reaction was consumed through RWGSR since more CO_2_ was generated from the samples with iron catalysts and iron is very active for the RWGSR under the process conditions. From H_2_ evolution trends, the catalytic activities of iron catalysts from different precursors were with the following descending order: Fe-N > Fe-S > Fe-Cl_3_ > Fe-Cl_2_. Methane was released from the decomposition of kraft lignin between 200 and 1000 °C under an argon atmosphere (Figure 2b). Methane released below 500 °C was mainly caused by the fragmentation of kraft lignin side chains. Demethylation of the aromatic methoxy groups (–O–CH_3_) also contributed to the methane formation in the low temperature range. Methane formation at the temperature above 500 °C was attributed to the breaking down of aromatic ring skeletons. Methane was observed in two temperature zones for Fe-N lignin samples, that is, the relative small and sharp peak at 250 °C and a wide and strong methane peak above 400 °C were observed. The methane evolution peak at a low temperature for Fe-N lignin sample tended to shift to the lower temperature compared to that of raw kraft lignin, that is, the first peak ranged from 455 °C to 240 °C and the high temperature methane evolution peak area was increased significantly. This might be because of the promotion effect of iron on lignin decomposition. The formation of methane significantly increased above 400 °C for Fe-N samples, possibly because of iron components catalytically cracking down aromatic ring skeletons in kraft lignin. Methane evolution profiles of FeSO_4_, FeCl_2_ and FeCl_3_ samples were similar to that of raw lignin but the methane evolution peaks of FeCl_2_ and FeCl_3_ samples at the low temperature shifted to lower temperatures compared to Fe-N sample, the first peak ranged from 455 °C to 250 °C for FeCl_3_ samples and 455 °C to 350 °C for FeCl_2_ samples. These shifts in temperatures attributed to the catalytic decomposition activity of iron ions (Fe^3+^ and Fe^2+^) to methoxy groups (–O–CH_3_). No significant difference in the temperature shift was observed for FeSO_4_ samples.

Two CO evolution peaks were observed for raw kraft lignin samples under an argon atmosphere. The CO evolution peak at a low temperature was 418 °C, which was mainly contributed to the decomposition of carboxyl (C=O) groups and the CO evolution peak centered at 770 °C was attributed to the cracking down of carbonyl (C–O–C). The CO formation of Fe-N lignin samples had three temperature zones, that is, a sharp peak centered at 237 °C, a wide flat peak at 640 °C and a strong CO evolution peak at 900 °C (Figure 2c). The CO evolution at 237 °C was contributed by the decarbonylation reaction of the C_3_ side-chains of lignin, which was catalytically decomposed at low temperature by Fe^3+^. The CO peak at 640 °C was because of the cracking down of carbonyl (C–O–C), while the CO peak at 900 °C was most likely because of the thermal cracking down of char residues. The CO evolution profiles of FeSO_4_, FeCl_2_ and FeCl_3_ samples were similar to the ones of raw lignin but the CO evolution peak of FeCl_3_ samples at the low temperature shifted to the lower temperature region, which was because of the influence of the catalytic activity of iron ions (Fe^3+^) and the decarbonylation reaction. No significant difference in CO formation was observed for FeSO_4_ and FeCl_2_ samples.

CO_2_ released during lignin decomposition because of the decomposition of carboxyl and ester groups of kraft lignin. Two significant peaks presented during the decomposition of kraft lignin under an argon atmosphere (Figure 2d). Carboxyl (–COO–) was considered to be predominantly responsible for the peak at the low temperature of 407 °C. The CO_2_ evolution at the high temperature of 642 °C was assigned to ester groups when the thermal process was under an inert atmosphere.

CO_2_ was also observed in three temperature zones for Fe-N lignin samples, that is, a sharp peak centered at 237 °C, a wide strong peak at 630 °C and a weak flat evolution peak at 870 °C. The formation of CO_2_ at 237 °C was contributed by decomposition of carboxyl (–COO–) and COOH groups in lignin which were catalytically decomposed by Fe^3+^ at a lower temperature. The CO_2_ peak at 630 °C was because of the cracking down of ester groups, while the CO_2_ peak at 900 °C was assigned to the thermal cracking down of char residues. The CO_2_ evolution profiles of FeSO_4_, FeCl_2_ and FeCl_3_ samples were similar to the one of raw lignin but the CO_2_ evolution peak of FeCl_3_ samples shifted to the lower temperature region because of the influence of the catalytic activity of iron ions (Fe^3+^) to the decarbonylation reaction.

The formation of phenols started with the dehydration of –OH groups in alkyl side chains of lignin basic units, followed by the cleavage of ether bonds between these units. The profile of phenols from the decomposition of kraft lignin under an argon atmosphere (Figure 2e) showed a phenol peak at 473 °C. Phenol formation peaks shifted to the low temperature region for Fe-kraft lignin samples.

The plot of the evolution of a typical aromatic compound of benzene from the thermal decomposition of kraft lignin samples (Figure 2f) showed that the evolution of benzene from the decomposition of kraft lignin under an inert atmosphere was detected over a wide temperature range from 528 to 938 °C, with a peak temperature at 697 °C. The formation of benzene in Fe-N samples was detected over two temperature zones: a weak flat peak in the temperature range from 220 to 380 °C and a strong wide peak in the temperature range from 510 to 1000 °C with the peak centered at 778 °C. Benzene was observed to be generated at a steady increasing level for the Fe-S sample when the heating temperature was above 500 °C. The evolution of benzene in Fe-Cl_3_ samples was detected over 520 °C with a wide flat peak in the temperature range from 520 to 1000 °C. Benzene was the only gas observed released from Fe-Cl_2_ samples at temperatures above 720 °C.

The evolution of methanol from raw kraft lignin samples heated under an argon flow was detected over a temperature range from 351 to 695 °C with a peak temperature at 494 °C (Figure 2g). The evolution profiles of methanol from Fe-lignin samples were significantly different than those of raw kraft lignin in intensity and temperature range. Methanol evolution peaks of Fe-lignin samples all shifted to the low temperature region and also had weaker intensity. This could be because the effect of the catalytic activity of iron and to the decomposition of aromatic methoxy groups (CH_3_O–) and aliphatic –CH_2_OH group in lignin. Light hydrocarbons (mainly methane) were the preferred products other than methanol for iron-promoted samples (Figure 2g).

When lignin was de-polymerized, sulfur can be present in many components of the gas phase, such as dimethylsulfide, carbonyl sulfide and hydrogen sulfide (H_2_S). The H_2_S evolution was monitored during the decomposition of kraft lignin samples under an argon atmosphere (Figure 2h). H_2_S formed in the temperature range from 210 to 646 °C, with a maximum value reached at 341 °C. H_2_S formation profiles in Fe-lignin samples were different with these in raw kraft lignin samples in terms of intensity and temperature range. The H_2_S evolution peaks of Fe-lignin samples were all in low intensity range. No H_2_S gas was detected in Fe-N samples. The H_2_S peak in Fe-S samples shifted to a high temperature zone of 350–650 °C with its peak temperature at 470 °C. H_2_S released as a flat peak between 308 °C and 690 °C in Fe-Cl_3_ samples, while a wide flat H_2_S evolution peak in a temperature range of 268–880 °C was observed in Fe-Cl_2_ samples. The sulfur content in kraft lignin samples seems to be absorbed completely (Fe-N) or partially (Fe-S, Fe-Cl_3_ and Fe-Cl_2_) by iron components during the thermal decomposition.

There were two formation zones of HCl released as a volatile from both FeCl_3_-and FeCl_2_-promoted kraft lignin samples. Fe-Cl_3_ samples had a HCl evolution peak centered at 256.6 °C within a low temperature range from 205 to 597 °C. The thermal decomposition of hexahydrate iron(III) chloride produced iron(III) oxide, hydrogen chloride and water. These reactions occurred at a temperature above 250 °C.

FeCl_3_ + H_2_O → FeOCl + 2HCl

2FeCl_3_ + 3H_2_O → Fe_2_O_3_ + 6HCl

HCl began to free from the samples at a high temperature of 623.3 °C due to the desorption of surface Cl atoms with the assistance of hydrogen atoms: H· + Cl· → HCl.

For the Fe-Cl_2_ sample, HCl was formed at higher temperature compared to the Fe-Cl_3_ sample, the HCl peak attributed to the hydrolysis of FeCl_2_ was centered at 333 °C with a temperature range of 249–793 °C:

FeCl_2_ + H_2_O → FeO + 2HCl

the formation of HCl started at a high temperature of 798.8 °C. The formation peaks were contributed by the desorption of surface chlorine with the assistance of hydrogen atoms: H· + Cl· → HCl.

Figure 2j shows the trends of NO_2_ and O_2_ during temperature-programmed decomposition of iron nitrate-promoted lignin samples. NO_2_ and O_2_ appeared between 160 and 350 °C with a peak temperature of 230 °C. This indicated that the thermal decomposition of iron(III) nitrate produced iron(III) oxide, nitric oxide and oxygen [20]:

4Fe(NO_3_)_3_ → 2Fe_2_O_3_ + 12NO_2_ + 3O_2_

Figure 2k shows the trends of SO_2_ and SO_3_ during temperature-programmed decomposition of FeSO_4_-promoted lignin samples. The anhydrous FeSO_4_ released sulfur dioxide and white fumes of sulfur trioxide, which left a reddish-brown iron(III) oxide [21]:

2FeSO_4_ → Fe_2_O_3_ + SO_2_ + SO_3_

The decomposition of sulfate initiated around 450 °C, formed a sharp peak at 500 °C.

### 3.2. Iron Catalyst Effects

***Product distributions****—*Table 1 summarizes the yields of three phases, gas, liquid (tars, condensable vapors, etc.) and solid (carbon and metal), produced during the catalytic graphitization of Fe-kraft lignin samples at 1000 °C under an argon atmosphere. The yields of solid carbon residues were 35.3%, 35.0%, 33.6% and 31.3% for Fe-Cl_2_, Fe-Cl_3_, Fe-S and Fe-N as iron sources, respectively. Thermally treated Fe-lignin samples with Fe-Cl_2_ and Fe-Cl_3_ as iron sources had lower conversion rates than one with Fe-N.

### 3.3. Solid Products Characterization

***Elemental analyses***—Table 2 summarizes the results of C–H–N–S–Cl elemental analyses performed for raw kraft lignin and thermally treated Fe-lignin samples. The weight contents of C, H, N and S in raw kraft lignin samples were 65.2%, 6.1%, 0.1% and 0.8%, respectively and no Cl was detected. For thermally treated Fe-lignin samples, Fe-N samples had the highest carbon content of 98.5 wt%, followed by Fe-S of 97.3%, Fe-Cl_3_ of 95.7% and Fe-Cl_2_ of 95.0%; hydrogen contents were 0.4%, 0.6%, 0.8% and 0.1% for Fe-S, Fe-Cl_3_, Fe-Cl_2_ and Fe-N samples, respectively. No nitrogen was found in thermally treated Fe-lignin samples. Sulfur contents in thermally treated Fe-lignin samples were 0.7%, 0.4%, 0.2% and 1.2% for Fe-S, Fe-Cl_3_, Fe-Cl_2_ and Fe-N samples, respectively. The mass contents of chlorine detected in both Fe-Cl_2_ and Fe-Cl_3_ samples were 0.8% and 0.5%, respectively.

***Surface area***—Table 3 summarizes BET surface areas measured on samples promoted with different iron compounds. The surface area value was in the range of 55–108 m^2^/g and ordered as Fe-N > Fe-S > Fe-Cl_3_ > Fe-Cl_2_ from high to low, that is, the sample prepared from iron nitrate had the highest surface area among four sample groups evaluated.

**Figure 3:**
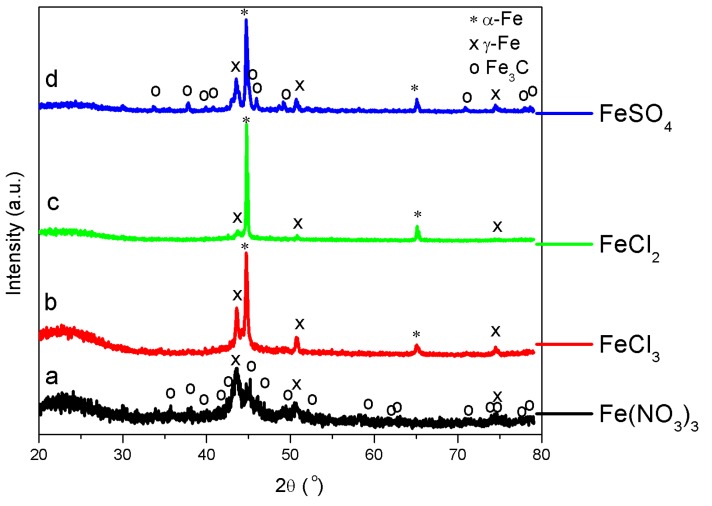
XRD patterns of Fe-lignin samples prepared using iron nitrate (**a**), iron(III) chloride (**b**), iron(II) chloride (**c**) and iron(II) sulfate (**d**) compounds.

***XRD***—Figure 3 shows the XRD patterns of thermally treated Fe-lignin samples prepared using Fe-N, Fe-S, Fe-Cl_2_ and Fe-Cl_3_ compounds. The symbols of *, x and o represent the characteristic diffraction peaks of metallic α-Fe (JCPDS, No. 87-0722), γ-Fe (JCPDS, No. 89-3939) and Fe_3_C (JCPDS, No. 89-2867), respectively. The diffraction peaks at 26.5° indicated the (002) plane of graphitic carbon. Both thermally treated Fe-lignin samples prepared using Fe-Cl_2_ and Fe-Cl_3_ exhibited sharp and narrow peaks of metallic iron (mainly α-Fe and γ-Fe). Thermally treated Fe-lignin samples prepared using Fe-S showed both metallic iron peaks (α-Fe and γ-Fe) and small and broad peaks of iron carbide (Fe_3_C). Thermally treated Fe-lignin samples prepared using Fe-N showed weak and broad peaks of metallic iron (mainly γ-Fe) and iron carbide (Fe_3_C), which indicated the presence of smaller Fe particles in Fe-N prepared samples compared to Fe-Cl_2_ and Fe-Cl_3_ prepared samples. The short-broad diffraction peaks observed in Fe-N prepared samples suggested good dispersion of iron species in the product. The sharper diffraction peaks of Fe-Cl_2_ and Fe-Cl_3_ prepared samples implied a growth in the crystallite size of metallic irons or iron carbide (Fe_3_C).

The mean particle size was calculated for the most intense diffraction peaks of α-Fe, γ-Fe and Fe_3_C using the Scherrer formula [15]. Only α-Fe and γ-Fe were detected in Fe-Cl_2_ and Fe-Cl_3_ prepared samples, while α-Fe, γ-Fe and Fe_3_C were observed in Fe-S and Fe-N prepared samples. The mean sizes of α-Fe nanoparticles were 79.6, 40.2, 59.1 and 18.5 nm for Fe-Cl_2_, Fe-Cl_3_, Fe-S and Fe-N prepared samples, respectively; while the γ-Fe grain sizes averaged 15.3, 30.2, 21.9 and 9.8 nm, respectively. The mean sizes of Fe_3_C nanoparticles were 22.5 and 19.7 nm for Fe-S and Fe-N prepared samples, respectively.

**Figure 4:**
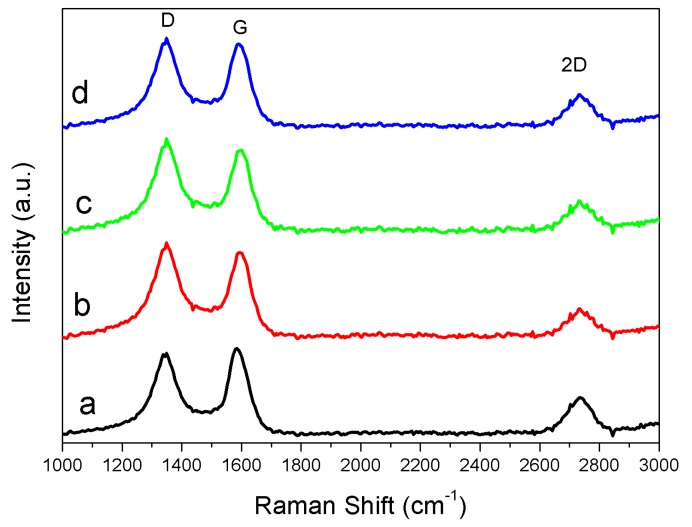
Raman spectra of thermally treated Fe-lignin samples prepared using different iron sources as catalysts: Fe-N (**a**), Fe-Cl_3_ (**b**), Fe-Cl_2_ (**c**) and Fe-S (**d**).

***Raman***—Figure 4 is the Raman spectra of thermally treated Fe-lignin samples prepared with four iron sources at 1000 °C, displaying a characteristic graphite G-band at 1580 cm^−1^, D-band at 1350 cm^−1^ and 2D-band at 2710 cm^−1^, respectively. The Raman spectra were fitted by Lorentz function to obtain values of I_D_, I_G_, I_D+G_ and I_2D_ values were calculated through fitting Raman spectra with the Lorentz function. The intensity ratios of the D to G band (I_D_/I_G_) of Fe-Cl_2_ and Fe-Cl_3_ prepared samples were 1.56 and 1.57, respectively, which were higher than those prepared with Fe-Sand Fe-N of values 1.43 and 1.29, respectively. These results suggested that thermally treated Fe-lignin samples prepared from iron sources of Fe-Cl_2_ and Fe-Cl_3_ had higher disorder structures, while Fe-N prepared samples had a higher graphitic degree.

**Figure 5:**
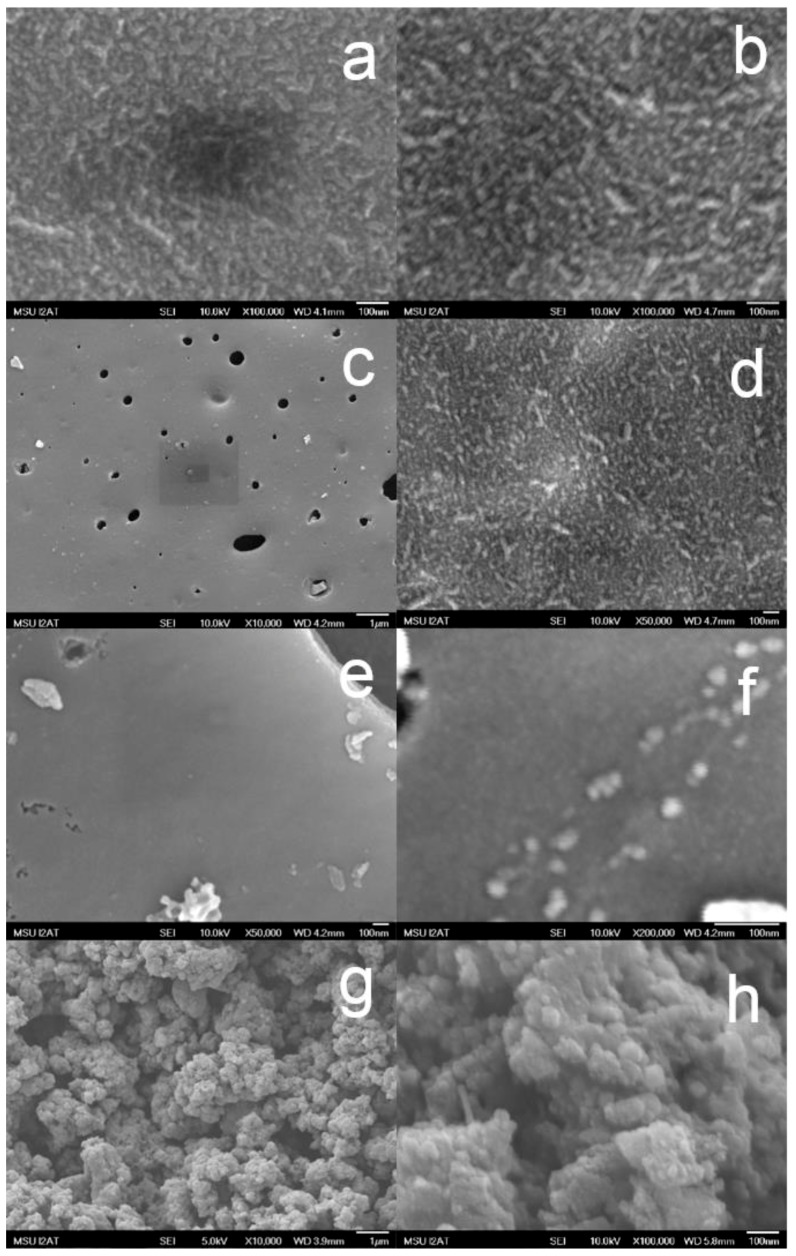
Scanning electron microscope images of four iron source (iron(II) sulfate (**a**,**b**) iron(II) chloride (**c**,**d**), iron(III) chloride (**e**,**f**) and iron nitrate (**g**,**h**)) promoted kraft lignin samples thermally treated at 1000 °C for 1 h under an argon flow.

***Scanning electron microscope (SEM)***—The SEM images (Figure 5) show typical morphologies observed on thermally treated Fe-lignin samples prepared with four iron sources of Fe-N, Fe-S, Fe-Cl_2_ and Fe-Cl_3_. The morphologies of Fe-Cl_2_, Fe-Cl_3_ and Fe-S prepared samples were significantly different from ones of Fe-N samples. The Fe-Cl_2_, Fe-Cl_3_ and Fe-S prepared samples had large pieces of solid grains. At the low magnification, the surfaces of Fe-Cl_2_, FeCl_3_ and Fe-O prepared samples were smooth and clean and with some gas channels (holes). At the high magnification, a layer of fine particle structures was observed over the surface of these samples. The morphologies of Fe-Cl_2_, Fe-Cl_3_ and Fe-S prepared samples looked more like raw lignin samples thermally treated at 1000 °C without any catalyst added.

The SEM images of Fe-N prepared samples showed a very fine powder structure, that is, small particles at the low magnification. At the high magnification, these particles were spherical nanoparticles with a relatively uniform particle size ranging between 5 and 10 nm. XRD results proved that these nanoparticles were composed of α-Fe, γ-Fe, iron carbide and graphene structures.

**Figure 6:**
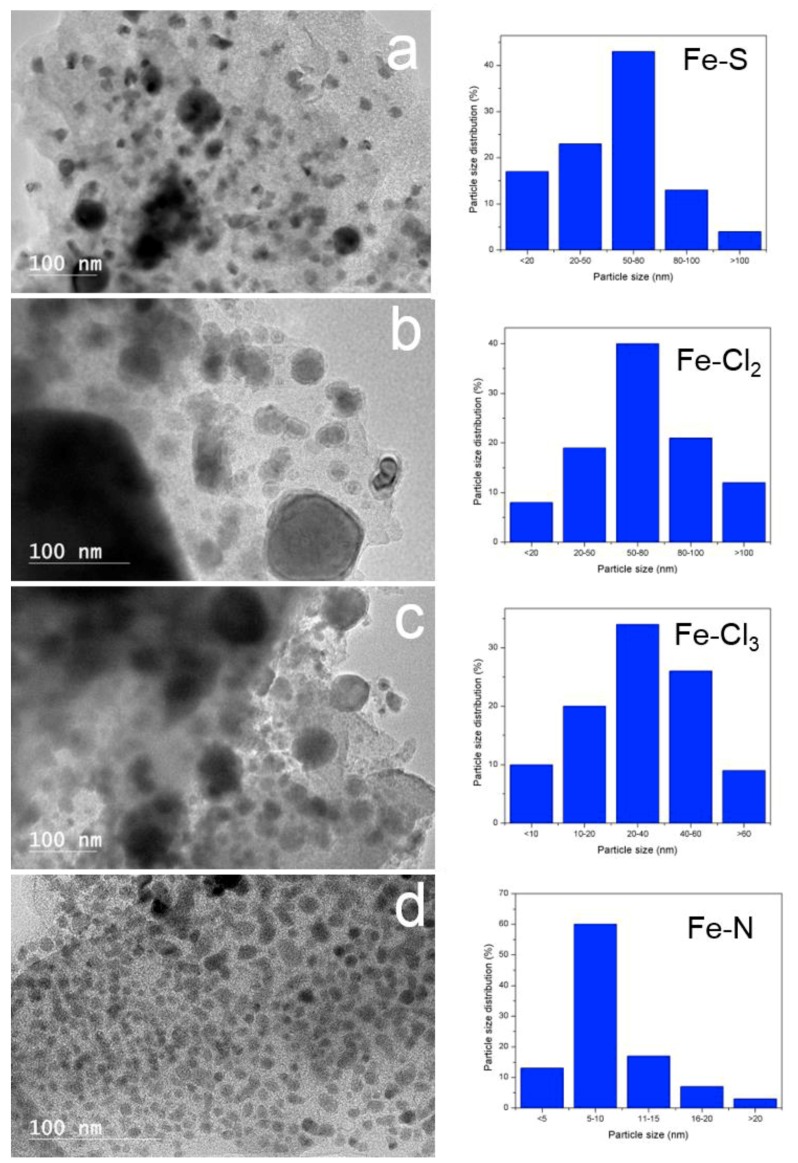
High resolution transmission electron microscope images of four iron sources (iron(II) sulfate (**a**) iron(II) chloride (**b**), iron(III) chloride (**c**) and iron nitrate (**d**)) promoted kraft lignin after thermal-treated at 1000 °C for 1 h under an argon flow.

***High resolution transmission electron microscopy (HRTEM)***—Figure 6 displays HRTEM images of thermally treated Fe-lignin samples. The dark spots in HRTEM images represented iron species dispersed in carbon-based materials. The nanoparticles in Fe-N prepared samples had core-shell structures with the diameter of core nanospheres in 3–5 nm range. The carbon shells exhibited ordered graphene plane structures. These Fe-core and graphene structure shelled nanoparticles homogenously embedded in the amorphous carbon framework (gray matrix). High-magnification TEM images indicated that these core-shell structures contained onion-like graphitic carbon nanostructures. The morphology of Fe-lignin samples prepared with iron chlorides and iron(II) sulfate was much different compared with Fe-N prepared samples. When iron chlorides and iron(II) sulfate were used as catalyst sources, serious agglomeration of iron particles occurred and the particle size reached as large as 50–100 nm in diameters. These iron nanoparticles unevenly distributed in the amorphous carbon matrix contained non-uniformly shaped solid iron crystallites. Most of iron-core nanoparticles in Fe-Cl_2_, FeCl_3_ and Fe-S prepared Fe-lignin samples were shelled with disordered amorphous carbon structures. This was presumably the seriously sintering of metallic iron particles because XRD results revealed that larger crystallite sizes of iron particles observed in Fe-Cl_2_, Fe-Cl_3_ and Fe-S prepared Fe-lignin samples than ones prepared with Fe-N. These results disclosed that uniformly dispersed smaller iron nanoparticles were beneficial for obtaining a highly performed iron catalyst towards the graphitization to kraft lignin, while agglomerated larger iron nanoparticles deteriorated the catalytic performance of irons as a catalyst. Variation in crystallite size of iron species with different iron catalysts was observed in the order of Fe-Cl_2_ > Fe-Cl_3_ > Fe-S > Fe-N.

## 4. Discussion

The elemental composition of lignin varies from species to species but in general, there are ~60% of carbon, 4–6% of hydrogen, ~28–31% of oxygen and 1% of ash [22]. Irons have shown a high activity as catalysts in catalytic conversion of solid carbon to graphite-based materials [23]. A major issue in catalytic graphitization of solid carbon sources including lignin is the contact degree between raw carbon materials and the catalyst [24]. When an iron salt is used as the iron source for a catalyst, the simple mixing of the iron salt dissolved in a solvent with lignin in the powder form is assumed that the iron can be easily distributed over the surface of lignin. This is because in general an iron salt has a relative low melting point. Therefore, dissolving the iron salt in a solvent can form a uniform solution. The initial distribution of an iron salt solution in lignin should be homogeneous, therefore, the iron as the catalyst can penetrate the lignin matrix under the catalytic thermal conditions and thus iron ions anchored at reacting carbon sites. This can be achieved only when a proper method is employed to achieve high iron dispersion over lignin.

Generally, an iron-solid biomass precursor is prepared using the impregnation method, that is, typically a dry biomass material such as lignin is added to the iron salt solution. After mixed, the iron salt solution is absorbed through the capillary forces inside the pores of the solid biomass material. Metal ions will further diffuse into biomass matrix and be anchored there through bonding to oxygen-containing groups in biomass.

Lignin is a 3-D complex organic polymer with huge molecules and while kraft lignin has a relative high hydrophobicity. Iron salt aqueous solution is repelled from kraft lignin molecules. It is very difficult for iron ions in an aqueous solution to penetrate into lignin particles. Using a traditional impregnation method will yield a poor contact degree of catalyst-lignin in general [25]. To uniformly blend iron salt with kraft lignin, iron-kraft lignin precursors were prepared in this experiment through a ‘co-precipitation’ technique, that is, both kraft lignin and iron salt first pre-dissolved in a solvent separately. Iron salts were dissolved in de-ionized (DI) water, while kraft lignin was dissolved in tetrahydrofuran (THF). Both solutions were then carefully mixed, iron salt and lignin would uniformly precipitate as a solid from the solvents. The iron-lignin precursors were achieved after vaporizing the solvents.

It was observed in this work that the catalytic performance in graphitization of kraft lignin was significantly affected by irons from different iron salts. The catalytic performance of four iron salts evaluated as iron sources for catalysts in this work was rated at the order of Fe-N > Fe-S > Fe-Cl_3_ > Fe-Cl_2_ from better to worse. The catalytic performance of a catalyst depends on the dispersion of the iron in the Fe-lignin system. The catalyst dispersion can potentially be improved by two methods in catalyst-lignin systems: (1) improving contact between lignin and catalyst during the initial stages of preparing a Fe-lignin precursor and (2) optimizing the physical properties of a catalyst, that is, increasing its surface area through reducing its particle size, or crystallite size [26]. However, sintering or agglomeration was observed for Fe-Cl_2_ and Fe-Cl_3_ prepared Fe-lignin samples under catalytic graphitization process conditions. Four different iron salts evaluated yielding different iron dispersions in lignin could be because of iron salt properties like iron salt solubility in solvent (usually water), ionized iron properties in water, iron salt melting temperature, iron salt decomposition temperature, iron-lignin bonding strength as well as anion properties in the iron salts. These parameters of iron precursor properties affecting the structure of iron-lignin precursors and final catalytic performance on graphitization of kraft lignin were discussed as followings.

### 4.1. The Solubility and the Hydration Properties of Iron Ions

The dispersion of iron ions in lignin and the formation of iron-lignin complex are typically the first step in the preparation of Fe-lignin precursors [9,10]. While the masses of Fe^2+^ and Fe^3+^ are practically the same but the ionic radii of the ions differ considerably (0.92 Å for Fe^2+^ and 0.79 Å for Fe^3+^) because of the different charge states. Fe^3+^ having a much higher charge density can substantially cause a different hydration behavior compared to Fe^2+^. Different hydrated complex iron ions will form when iron salts solvate in water [27]. In Fe (NO_3_)_3_ aqueous solution, iron ions mainly exist as the octahedral hexa-aqua complex [Fe (H_2_O)_6_]^3+^ [28,29]. Similar to that of iron nitrate, the iron(II) sulfate dissolves in water to give the similar aquo-complex [Fe (H_2_O)_6_]^2+^, which has octahedral molecular geometry. For the FeCl_2_ aqueous solution, besides the octahedral hexa-aqua complex [Fe(H_2_O)_6_]^2+^, several chloro-complex species are identified in acidic solutions of ferrous chloride [30], which are: octahedral monochloro-complex [Fe(H_2_O)_5_Cl]^+^, dichloro-complex [Fe(H_2_O)_4_Cl_2_] and tetrahedral tetrachlorocomplex [FeCl_4_]^2−^. The latter species, however, is formed exclusively at very high chloride excesses and/or high temperatures. Similarly, the complexes formed by ferric chloride (FeCl_3_) are: octahedral hexaaqua complex [Fe(H_2_O)_6_]^3+^, monochlorocomplex [Fe(H_2_O)_5_Cl]^2+^, dichloro-complex trans-[Fe(H_2_O)_4_Cl_2_]^+^, trichloro-complex [Fe(H_2_O)_3_Cl_3_] and tetrahedral tetrachlorocomplex [FeCl_4_]^−^. Correspondingly to the ferrous salt, the highest order chloro-species is only found at high chloride excesses and/or high temperatures. These solvated iron ions have different charge density and radius. Of these solvated iron complexes, the octahedral hexa-aqua complex [Fe (H_2_O)_6_]^3+^ has the highest charge density and smallest ionic radii, while the chlorocomplexes have a relative lower charge density and larger ionic radii. The higher charge density and smaller ionic radii, the stronger tendency of iron ions to bonding with oxygen-containing groups in lignin when they are contacting. Therefore, it is easy for [Fe(H_2_O)_6_]^3+^ to penetrate onto lignin molecules and to be uniformly distributed in catalyst-lignin precursors. While the links between chlorocomplexes and lignin are weak and the distribution of these chloride-containing iron precursors is poor in lignin.

### 4.2. Thermal Stability of Iron Precursors

The decomposition of Fe-lignin precursors is the next step (after dispersion of Fe ions onto lignin and drying) before the graphitization of kraft lignin. The decomposition of Fe-lignin precursors usually results in the formation of iron oxide species which can be converted to the active iron metal phases in kraft lignin matrix. Previous literature works [31] suggested that the decomposition of metal precursors at milder conditions generally leads to a higher metal dispersion. This means that iron salt precursors decomposed at a lower temperature will result in better iron dispersion and catalytic performance. The iron salts investigated in this work are Fe (NO_3_)_3_, FeSO_4_, FeCl_3_ and FeCl_2_. Of these iron salts, iron nitrate will be easily broken between 100–250 °C, release gaseous H_2_O, HNO_3_, NO and NO_2_ and leave Fe_2_O_3_ as solid residues. When heated, iron(II) sulfate first loses its water of crystallization and original crystals are converted into an anhydrous solid [32]. The TGA curves of Fe-S prepared samples showed the initial dehydration of FeSO_4_·7H_2_O occurred at the temperature up to 200 °C (FeSO_4_·7H_2_O → FeSO_4_ + 7H_2_O). When further heated, the anhydrous material decomposes and releases sulfur dioxide and sulfur trioxide, leaving a solid iron(III) oxide [33]. The decomposition of sulfate initiates around 500 °C. TGA curve demonstrated a sharp decrease in weight at 500 °C for Fe-S prepared samples, followed by a slower decomposition at 500–600 °C. Other researchers observed a sharp decrease in weight because of the decomposition in the temperature range of 550–625 °C for FeSO_4_ prepared samples.

At the higher temperature of 700 °C, anhydrous FeCl_3_ decomposes and forms ferrous chloride [34].

FeCl_3_ above 700 °C → 2FeCl_2_ + Cl_2_

But Fe(III) chloride hydrate (FeCl_3_·xH_2_O) can proceed simultaneously by both dehydration and hydrolysis at the temperature above 100 °C (FeCl_3_ + 2H_2_O → Fe(OH)_2_Cl+ 2HCl or FeCl_3_ + 3H_2_O → Fe(OH)_3_ + 3HCl). In the temperature range of 250–300 °C, a stable hydrated Fe (OH)_2_Cl is formed [33]. Around 400 °C, Fe(OH)_2_Cl continues to decompose and form mostly Fe_2_O_3_, which however retains some OH groups and Cl− ions [35].

Similar to FeCl_3_, FeCl_2_ (anhydrous) itself is thermally stable under temperatures up to 1000 °C, its hydrate (FeCl_2_·xH_2_O) can undergo partial hydrolysis or thermal decomposition to Fe_3_O_4_, HCl and H_2_ (reaction: 3FeCl_2_ + 4H_2_O → Fe_3_O_4_ + 6HCl + H_2_) between 550 and 800 °C [36].

TGA and TPD-MS results illustrate that Fe-N prepared samples decompose at 237 °C, which is the lowest among four iron salts evaluated, that is, 300 °C for Fe-Cl_3_, 323 °C for Fe-Cl_2_ and 449 °C for Fe-S prepared samples. The lower iron nitrate decomposition temperature enhances iron dispersion, which is consistent with the XRD (Figure 3), SEM (Figure 5) and TEM (Figure 6) results.

### 4.3. Interaction of Iron with Functional Groups in Kraft Lignin

The effects of iron precursors on solid carbon yields during de-polymerization and initial carbonization may also be explained by considering the association of Fe with the lignin structure when preparing Fe-lignin precursors. Fe ions (Fe^3+^ and Fe^2+^) may be coordinated to a series of oxygen-containing ligands in lignin including O^2^^−^, OH^−^, H_2_O, −C=O, C−O−C and –COO–. Thus, Fe in lignin will greatly change the macromolecular structure of lignin by bringing the coordination sites closer and making the lignin structure tighter. Firstly, irons stabilize the oxygen-containing functional groups (such as carboxylic groups) which they are linked with, leading to increased contact degree between Fe as the catalyst and lignin. Secondly, irons enhance the thermal cracking down and de-polymerization of lignin, yielding the remove of most of oxygen and hydrogen via catalytic thermal process and generating solid carbon structures around iron particles. During the catalytic thermal process, the bonding between Fe ions and lignin structure will break down and iron ions would be converted into metallic or other forms with increasing temperature. The carbon structures around iron particles will also further decompose to produce more gases. The iron had different effects on the gas production. The gas fraction was 44.8%, 45%, 46.3% and 50.7% for Fe-Cl_3_, Fe-Cl_2_, Fe-S and Fe-N prepared samples, respectively. The H_2_S evolution profiles (Figure 2h) showed no H_2_S detected in Fe-N samples and also elemental data proved the highest sulfur content in Fe-N samples. These further proved the highest dispersion iron in Fe-N samples, which would simultaneously absorb H_2_S released from kraft lignin structure during the catalytic decomposition process.

### 4.4. Impacts of Chlorine on Iron Catalyst

Chlorine is considered to be a very strong poison to metal catalysts, particularly in transitional metal catalysts for F-T process [37] and ammonia synthesis [38]. A significant amount of residual Cl^−^ ions (Table 2) was observed in Fe-Cl_2_ and Fe-Cl_3_ prepared samples during the catalytic decomposition process (Figure 2i). The presence of chlorine in Fe-lignin samples could strongly alter iron catalytic properties. Residual chlorine, which remained in the catalysts after the thermal decomposition, can have several impacts on the properties and performance of the catalysts [39]. First, the existence of chloride on metal surfaces will accelerate the agglomeration/sintering of metal particles [40]. This one might relate to the formation of iron-chlorine compounds on iron particle surfaces. Iron particles with a layer of Fe-Cl on surfaces have a tendency to merge and agglomerate to form larger size particles. This phenomenon could cause an increase in the size of iron crystallites and thus, a decrease of the surface area and iron dispersion. The second possible mechanism is also based on Fe-Cl formation. The formation of Fe-C phases between the active iron metal and the carbon species can be affected by the presence of chlorine. Iron-chlorine compounds are very stable at a high temperature, a thin film of FeCl_2_ on metal iron surfaces formed when a molecular beam of carbon tetrachloride bombarded on an iron film surface in ultrahigh vacuum at 780 °C [41]. The formation of FeCl_2_ thin film will be a diffusion barrier for preventing carbon atoms to be transferred into and interact with iron particles and then the growth of graphene structure can be stopped. Third, experimental results indicate that the formation of graphene structures from solid carbon sources is partially related to the carbonaceous gases (such as CH_4_ or CO) generated from the decomposition of solid carbon feedstock. Residue chloride adsorbed on catalyst surfaces will deactivate iron active sites by preventing the adsorption of carbonaceous gas molecules [42].

## 5. Conclusions

In the present study, the effect of irons containing different anions like nitrate and chloride on the catalytic thermal graphitization of kraft lignin to graphene-based materials was investigated. Four iron salts (iron(III) nitrate, iron(II) chloride, iron(III) chloride and iron(II) sulfate) were used as iron sources. The catalytic decomposition of iron-promoted lignin samples was examined using TGA and TPD experiments. The thermally treated Fe-lignin samples were measured and characterized by elemental analysis, XRD, Raman, SEM and HRTEM. It was found that four different iron salts influenced the states and dispersion of iron species in Fe-lignin samples. Among four iron salts studied, iron nitrate was the most active one, while both the iron(III) chloride and iron(II) chloride showed poor activity. The residual chlorine presented over iron surfaces might prevent the interaction of iron with functional groups in lignin, decrease iron dispersion and reduce activity of irons as the catalyst. The better activity on the catalytic decomposition of iron nitrate-lignin could be attributed to the highly dispersed iron nanoparticles and stronger iron-lignin interaction. The use of iron(III) nitrate as an iron source for the catalyst for iron-kraft lignin precursors gave higher iron dispersions than the catalysts prepared from iron(II) chloride and iron(III) chloride.

## Figures and Tables

**Table 1:** Effects of iron sources on products weight percentage distribution of catalytic decomposition of kraft lignin at the temperature of 1000 °C for 1 h.

Fe Resources	Solid Carbon	Liquid	Gas
Fe-S	33.6	19.5	46.9
Fe-Cl_3_	35.0	20.2	44.8
Fe-Cl_2_	35.3	19.7	45
Fe-N	31.3	18	50.7

**Table 2:** Weight percentage values of elemental analyses of raw kraft lignin and thermally treated Fe-lignin samples with different iron catalysts under argon atmosphere at 1000 °C for 1 h.

Samples	C	H	N	S	Cl
Raw kraft lignin	65.2 ± 0.2	6.1 ± 0.2	0.1 ± 0.05	0.8 ± 0.2	N/A
Fe-S	97.3 ± 0.5	0.4 ± 0.2	N/A	0.7 ± 0.1	N/A
Fe-Cl_3_	95.7 ± 0.9	0.6 ± 0.3	N/A	0.4 ± 0.1	0.5 ± 0.3
Fe-Cl_2_	95.0 ± 0.7	0.8 ± 0.2	N/A	0.2 ± 0.2	0.8 ± 0.4
Fe-N	98.5 ± 0.5	0.1 ± 0.05	N/A	1.2 ± 0.3	N/A

**Table 3:** Surface area (S_g_) of carbon-based nanomaterials from kraft lignin promoted with different iron catalysts.

Samples	S_g_ (m^2^ g^−^^1^)
Fe-N	108
Fe-Cl_2_	55
Fe-Cl_3_	60
Fe-S	79
